# Modeling of an Optically Heated MEMS-Based Micromechanical Bimaterial Sensor for Heat Capacitance Measurements of Single Biological Cells

**DOI:** 10.3390/s20010215

**Published:** 2019-12-30

**Authors:** Abdullah Alodhayb

**Affiliations:** 1 Research Chair for Tribology, Surface, and Interface Sciences, Department of Physics and Astronomy, College of Science, King Saud University, Riyadh 11451, Saudi Arabia; aalodhayb@ksu.edu.sa; 2 Aramco Laboratory for Applied Sensing Research, King Abdullah Institute for Nanotechnology, King Saud University, Riyadh 11451, Saudi Arabia; 3 Department of Chemical and Biological Engineering, University at Buffalo, The State University of New York, Buffalo, NY 4260, USA

**Keywords:** bimaterial microcantilever, optical heating, thermal analysis, MEMS-based calorimeter

## Abstract

Detection of thermal activities of biological cells is important for biomedical and pharmaceutical applications because these activities are closely associated with the conformational change processes. Calorimetric measurements of biological systems using bimaterial microcantilevers (BMC) have increasingly been reported with the ultimate goal of developing highly sensitive and inexpensive techniques with real-time measurement capability techniques for the characterization of dynamic thermal properties of biological cells. BMCs have been established as highly sensitive calorimeters for the thermal analysis of cells and liquids. In this paper, we present a simulation model using COMSOL Multiphysics and a mathematical method to estimate the heat capacity of objects (treated here as a biological cell) placed on the surface of a microcantilever. By measuring the thermal time constant, which is obtained from the deflection curve of a BMC, the heat capacity of a sample can be evaluated. With this model, we can estimate the heat capacity of single biological cells using a BMC, which can potentially be used for the thermal characterization of different biological samples.

## 1. Introduction

Considerable ongoing research has been devoted for the development of simple and cost-effective sensor systems for biological applications [1,2,3,4,5,6,7,8,9,10]. The detection of thermal activities of biological cells is of particular interest [11,12,13,14,15]. Measurement and temperature control of biological cells are considerable contributors to the development of new research methods in the study of genetics and the development of diseases, and they provide further insight into the behavior of cancerous tumors [16,17]. Because the generation of heat in cells is a function of the amount of energy consumed, analysis of heat generation is necessary for studying cell behavior. Single cell thermal measurements are frequently performed in bulk conditions in which averaged data for a group of cells (~105 cells) are used to represent the activity of a single cell [18,19]. This results in imprecise measurements of the caloric output of single cells, which can lead to several confounding factors about the measurements [11]. Averaged data from a large group of cells make understanding the mechanism of heat generation by individual cells very difficult. Consequently, the detection of metabolic release within single cells and not from the average measurement of a group of cells can considerably reduce research costs, time, and the number of animal test subjects used in experiments designed to detect and diagnose abnormal cell types. Therefore, such thermal measurements of single cells are key in the fields of medical science and biotechnology [20,21].

Several techniques have been demonstrated for heat measurements of single cells such as hydrophilic fluorescent nanogels [22], thermocouples (TC) [17], Q-dots [23], multiwalled carbon nanotubes (MWCNTs) [24], thermosensitive dyes [25], and green fluorescent proteins (GFPs) [26]. These techniques have high sensitivity (temperature resolution of 1.8 mK is acquired [21]); however, they require the introduction of a thermometer into the cell. Moreover, alternative methods have also been proposed in which microfabricated thermal nanocalorimeter systems are used to measure heat generated by single cells [26,27]. Such techniques require the immersion of cells in water, thereby reducing the sensitivity of systems because of liquid damping. To try to overcome the limitation associated with immersing cells in water, microfluidic calorimeters have increasingly been employed for heat measurements of single cells [11,28]. The measurement principle of microfluidic calorimeters depends on measuring the resonance frequency of the resonator in response to the heat generated by a cell attached to the sample stage. Such measurements take place in a vacuum, which ensures minimal heat loss and damping. Calorimeters based on microcantilever sensors have also gained considerable interest because of their high sensitivity and simplicity [29,30,31]. The most common configuration of microcantilever calorimeters is the bimaterial cantilever, which is a composite structure formed by depositing thin layers of silicon nitride and a metal (aluminum or gold). Despite the fact that gold is extensively used in cantilever sensing applications, an aluminum–silicon nitride combination is preferred in thermal sensing because the thermal expansion coefficient of aluminum is higher than that of gold. Small temperature variations cause a bimaterial microcantilever (BMC) to undergo a mechanical deflection because of the different thermal expansion coefficients of the constituent layers. Because of the ultrasensitivity of BMCs, they have been used as sensing platforms in many applications [32,33]. Toda et al. reported a highly sensitive bimaterial microcantilever temperature sensor [34]. They successfully detected, in situ, the local heat generated by a single mammalian cell. Research efforts toward developing BMCs have employed nanomechanical calorimetric infrared (IR) spectroscopy in a wide range of chemical and biological applications [35,36]. In this technique, a BMC undergoes a mechanical deflection in response to the heat produced by the IR radiation. Khan et al. demonstrated the use of a BMC as a closed-chamber calorimeter that is optically heated with IR radiation to successfully measure the heat capacity of five volatile organic compounds (VOCs) with varying thermal outputs [37]. The reported system was able to provide online thermal characterization of small volumes of liquids with a resolution of 23 mJ/(gK). This system combines the high thermal sensitivity of a BMC and optical heating by IR radiation, which overcomes the limitation of resistive heating, and shows the high potential of BMCs for sensitive thermal measurements. BMCs are known to have excellent thermal sensitivity with a temperature resolution of 10^−5^ K and power of 40 pW [38]. To gain a better understanding of the mechanism of a BMC during optical heating and to extend the use of optical heating of BMCs to measure the heat capacity of single cells, we have developed a simulation model using COMSOL Multiphysics, which is used to measure the time constant of BMC deflection. Heat capacity measurements of single cells are very important in understanding thermal dynamics in biological cells and for processes such as conformational changes [39]. In this model, a cell (i.e., a yeast cell) is placed on the middle of a cantilever and is subjected to IR radiation. This step is preceded by investigating the response of the bimaterial cantilever before the sample is placed. Because the time constant of the deflection can be obtained from the thermal response of the cantilever, measuring the heat capacity of samples placed on its surface is possible using the mathematical model developed by us. This model is very important for predicting the cantilever deflection in response to the optical heating of the cell on its surface. Therefore, it becomes a means of estimating dynamic thermal properties such as heat capacity and thermal conductivity.

## 2. BMC Structure and Theory

**Figure 1:**
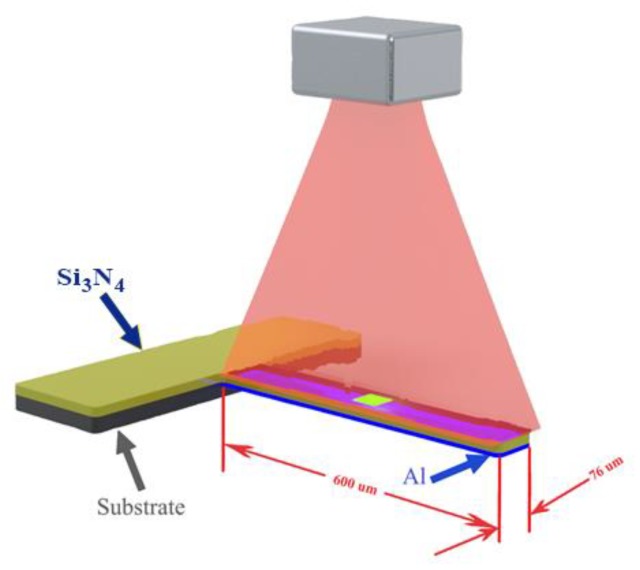
A schematic representation of the biomaterial microcantilever used in this model. IR light is provided from top and the sample is placed on the middle of the bimaterial microcantilever (BMC).

As stated earlier, the temperature variation causes the BMC to deflect because of the differential thermal stress caused by the difference of the thermal expansion coefficients of the constituent materials of the beam. The thermal response of the BMC can be analytically expressed by [29]:

(1) $$\frac{d^{2}z}{{\mathrm{dx}}^{2}}=6\left(\alpha_{1}-{\;\alpha }_{2}\right)\left(\frac{t_{1+}t_{2}}{t_{2}^{2}K}\right)\left[T\left(x\right)-T_{0}\right],$$

where

(2) $$K=4+6\left(\frac{t_{1}}{t_{2}}\right)+4{\left(\frac{t_{1}}{t_{2}}\right)}^{2}+\frac{E_{1}}{E_{2}}{\left(\frac{t_{1}}{t_{2}}\right)}^{3}+\frac{E_{2}}{E_{1}}\left(\frac{t_{2}}{t_{1}}\right),$$

where *z* is the vertical defection at a location *x* along the length of the cantilever; $\alpha_{1}$ and $\alpha_{2}$, and $t_{1}$ and $t_{2}$ are the coefficients of thermal expansion and the thickness of the constituting materials, respectively. T(x) and $T_{0}$ are the temperature profile of the cantilever along its length and the temperature of the cantilever at zero deflection, respectively. *E* is the Young’s modulus. The dimensions of the BMC used in this model were 600 μm in length, 76 μm in width, and 1 μm in thickness, as shown in Figure 1. The structural material of the BMC is silicon nitride, Si_3_N_4_, which has good thermal conductivity and high rigidity, making it an appropriate material for thermal analysis. The bimetallic structure is obtained by depositing aluminum on the bottom side with a thickness of 500 nm.

## 3. Generation of Numerical Data

**Figure 2:**
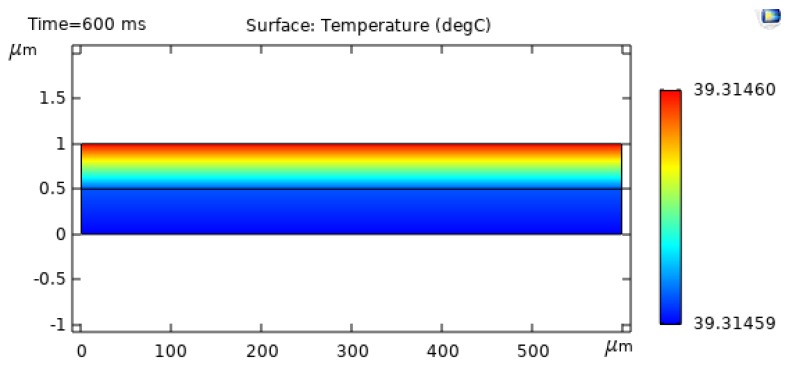
Temperature profile of the BMC.

In the two-dimensional model, the initial simulations were conducted only on the bimaterial microcantilever without any sample placed on the top. Such attempts were made to find the BMC deflection in response to the optical radiation. The top side (Si3N4) was irradiated with a comparatively large-diameter laser to the extent that the irradiation was considered uniform over the length and width of the cantilever beam. A laser of 635 nm wavelength was used in this study. The surface absorption was assumed to be 500 W/m^2^. There are several factors which affect the surface absorption, such as wavelength absorption by the material, the optical energy, the wavelength of light, and the reflectivity of the microcantilever. Uniform natural convection and radiative emission was observed from all the surfaces. A numerical simulation with COMSOL resulted in a temperature profile over the surface as shown in Figure 2.

As Figure 2 shows, the temperatures vary by only 1 °C in the fifth place. Therefore, the BMC may be considered a “lumped mass” at constant spatial temperature. This is due to the very small Biot number, arising from the small thicknesses and the relatively high thermal conductivity [38]. Transient heat transfer to and from a body depends on the relative internal and external resistance. Thus, the ratio of resistances for an object of size *D* may be stated as

(3) Bi=1ksD1h=hDks

where *k_s_* is the solid conductivity *h* is the convective heat transfer coefficient, and *Bi* is the Biot number. Whenever *Bi* << 1, or the internal resistance is considerably smaller than the external resistance, then the internal temperature gradient can be ignored in the, so called, “lumped mass” approximation. Typically, for free convection, *h*~20 W/m^2^K, and for a yeast sample of *D*~5 µm, with *k_s_*~0.03 W/mK we find that *Bi*~0.003, which is considerably lesser than 1, and which is typical for small objects.

The net heat transfer to/from a lumped mass is given by

(4a) $$Q_{net}=\left(q_{s}-q_{h}\right)A=\rho VC_{p}\frac{dT_{s}}{dt},$$

where *A* is the area, *V* is the volume (*V* = *HA*), and *T_s_* is the solid temperature. The (laser) heat source is $q_{s}$ and the convective loss is $q_{h}$. Thus, we can express:

(4b) $$\frac{\rho HC_{p}}{h}\frac{dT_{s}}{dt}+\frac{q_{h}}{h}=t_{c}\frac{dT_{s}}{dt}+T_{s}-T_{amb}=\frac{q_{s}}{h},$$

where $t_{c}$ is the time constant $t_{c}=\frac{\rho HC_{p}}{h}$, where ρ is the density, H is the height or characteristic length, and *h* is the heat transfer coefficient. The solution of Equation (4b) is

(5) $$T_{s}=T_{amb}+\frac{q_{s}}{h}+\left(T_{s}\left(0\right)-T_{amb}-\frac{q_{s}}{h}\right)e^{-t/t_{c}}.$$

**Figure 3:**
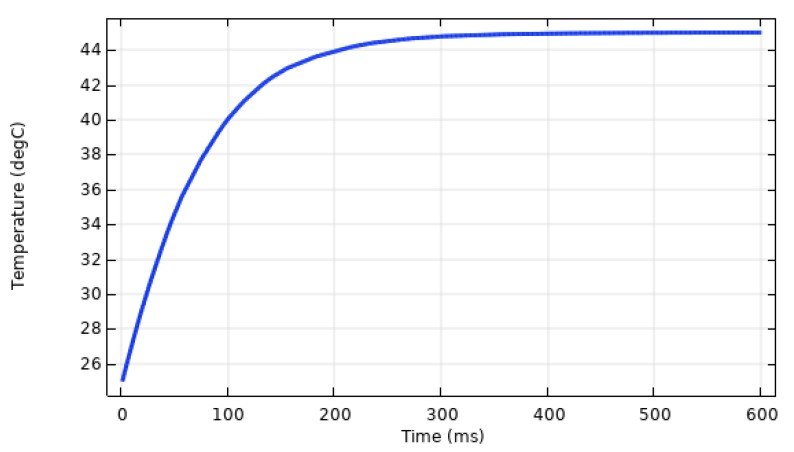
The temperature variation with time.

Because of the uniform heat absorption, and the “lumped mass” thermal characteristic, the uniform temperature increases at the same rate as the deflection, as shown in Figure 3, and rises to the maximum in approximately 300 ms, which is because of the linearity of the thermal expansions. The delay in the temperature increase is due to the specific heat, $C_{p}$, or thermal inertia of the materials. It is also important to mention that because of the small size of the cantilever and very minute temperature gradient, the convective and radiative heat losses have been neglected.

**Figure 4:**
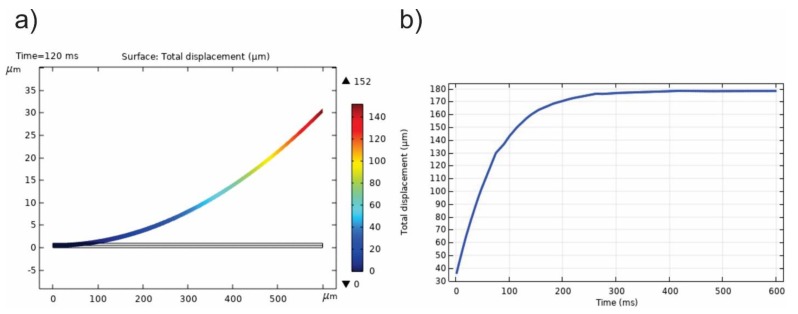
(**a**) BMC deflection in response to optical heating; (**b**) numerically generated-data showing the BMC displacement as a function of time.

Here the BMC is considered horizontal, to the extent that there is convective heat transfer from the tip to the anchor of the cantilever at the top surface. The convection coefficient *h* is assumed to be constant (*h*~20 W/m^2^K), irrespective of the temperature and the location on the BMC. In experiments, measuring temperature responses is difficult because of the small size. However, because of the small size this is difficult to do. Instead, the deflection of the tip of the cantilever can be measured. As laser heat is absorbed in the numerical model, the differential expansion causes the beam to deflect, after an irradiation time of 500 ms, and with a maximum deflection of 178 μm at the tip, as shown in Figure 4. These data that would usually be values measured in an experiment, but in this paper they are numerically generated.

## 4. Results and Discussion

The fact that a lumped-mass system has an exponential solution is well known [40]. Because this is a lumped-mass system, and the tip displacement is proportional to temperature, the y-displacement can be written as:

(6) $$v=v_{\infty }-\left(v_{\infty }-v_{0}\right)e^{-t/\tau }.$$

**Figure 5:**
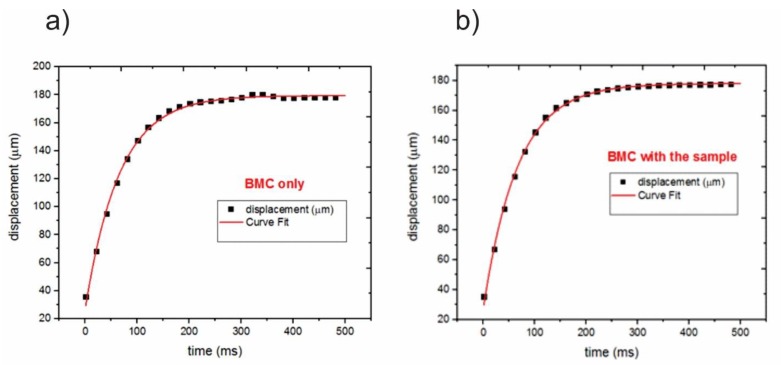
(**a**) Displacement and the curve fitting of the BMC before placing the sample; (**b**) displacement and the curve fitting for the BMC loaded with the sample.

By least squares regression these parameters were determined from the data in Figure 5a as $v_{\infty }$ = 178.7 μm, $v_{0}\;$= 27.1, and τ = 66.5 ms.

In experiments, the data would be measured (not generated by COMSOL), and the fitting would be regarded as a calibration of the microcantilever instrument. As demonstrated in Figure 5, there is an excellent agreement between the numerically generated data and the exponential function.

Subsequently, the displacement with the sample attached to the top surface was measured. The Irradiation and environs would be the same as the calibration (and the sample temperatures were still within 0.1 °C of the beam temperatures). The simulation sample, corresponding to a yeast cell with dimensions of 5 μm long and 2 μm high, was located in the axial center of the beam. In this case, the fitted parameters were $v_{\infty }$ = 177.1 μm, $v_{0}$ = 26.6, and τ = 69.3 ms, with excellent results, as seen in Figure 5b.

Because of the added thermal capacitance from the sample, the response of the total system should be somewhat slower and, therefore, yield a larger time constant when data are fitted, as seen.

For combined masses (or volumes) of the beam (B) and the sample (S), the total heat per unit volume (T) is given by

(7a) $${\left(\rho C_{p}\right)}_{T}=\frac{{\left(\rho C_{p}\right)}_{B}V_{B}+{\left(\rho C_{p}\right)}_{S}V_{S}}{V_{B}+V_{S}}.$$

The ${\left(\rho C_{p}\right)}_{T}$ and ${\left(\rho C_{p}\right)}_{B}$ values are obtained from measured data and curve-fitting, as mentioned above, and therefore, ${\left(\rho C_{p}\right)}_{S}$ is obtained from the inverse of (7a):

(7b) $${\left(\rho C_{p}\right)}_{S}V_{S}={\left(\rho C_{p}\right)}_{T}\left(V_{B}+V_{S}\right)-{\left(\rho C_{p}\right)}_{B}V_{B},$$

where *V* denotes volumes. Here ${\left(\rho C_{p}\right)}_{S}$ is obtained from the difference between two comparatively large numbers of nearly equal magnitudes which may be prone to some errors or uncertainties

The time constant for a convectively cooled lumped mass is given by

(8a) $$\tau =\frac{\rho C_{p}L_{c}}{h}\;\mathrm{or}\;\rho C_{p}=\tau h/L_{c},$$

where *L_c_* is the characteristic heat transfer length for the mass. Because *h* is the same in both cases, the time constants from (7b) are represented as:

(8b) $${\left(\frac{\tau }{L_{c}}\right)}_{S}V_{S}={\left(\frac{\tau }{L_{c}}\right)}_{T}\left(V_{B}+V_{S}\right)-{\left(\frac{\tau }{L_{c}}\right)}_{B}V_{B}.$$

The characteristic length may be estimated as *L_c_* = Vol/Area, which for the beam is the thickness, 1.0 μm; similarly, for the sample, *L_c_* = 1.1 μm. Hence, from Equation (8b) we can write

(8c) $$\tau_{S}=\frac{1.1}{1.0}\left\{\tau_{T}+V_{B}/V_{S}\left(\tau_{T}-\tau_{B}\right)\right\},$$

where *V_B_/V_S_* = 34. Therefore, the thermal time constant of the sample is *τ_s_* = 230 ms. The input data for the sample using numerical modeling is given by Equation (8a). After finding the thermal time constant of the sample, we measured the heat capacity of the sample which we found to be 2.165 kJ/kg·K. This value is in an excellent agreement with the literature value of yeast heat capacity, which is 2.17 kJ/kg·K [41]. This clearly shows that our model can be accurately used to estimate the heat capacity of samples placed on the top of the cantilever. Further attempts were made to investigate the effect of the position of the sample on the BMC surface. We moved the sample from the middle of the BMC to the top; however, no effect on the response was found. We however found no effect on the response. In fact, the thermal time constant obtained was almost similar to the case where the sample was placed in the middle. This feature is promising because in real experiments manipulating the position of the sample on the beam is very difficult. Therefore, our findings suggest that the sample may be placed anywhere between the middle and the top of the beam, and similar results will be obtained. The numerical model developed in this study to predict the static behavior of the BMC can be extended to be used to characterize thermal properties of chemical materials. The significance of this work also stems from the fact that the use of IR radiation has been found to greatly increase the intrinsic chemical selectivity of microcantilever sensor, which was one of the major limiting factors of microcantilever commercialization [42,43]. Despite the increasing number of literature reports on using photothermal spectroscopy techniques [44,45], there is still a need for more numerical studies such as the work presented in this study. Such numerical models would significantly help to gain more insight into the behavior of the BMC before an actual experimental is performed. Thermal analysis using bimaterial microcantilevers has also been a key factor in developing a better understanding about the BMC response during experiments. For example, the mass evolution that occurs during sensing experiments and results in a resonance frequency shift of the BMC can be better understood when thermal analysis is used [46,47]. Finally, we postulate that the results of this work can constitute a foundation to predict the BMC behavior and thus optimize the conditions before experiments are conducted.

## 5. Conclusions

In this work, a micromechanical bimaterial microcantilever (BMC) has been devised in which the heat capacitance of a biological sample can be determined from deflection measurements. A numerical thermal/structural model was constructed which indicated uniform time-varying temperatures under constant irradiation. This renders the device a, so called, lumped mass that results in exponential solutions. Numerically generated deflection data were used with regressions of exponential solutions to derive system time constants. From these values, the sample heat capacity was determined. The model developed in this paper is very useful for the thermal characterization of the samples. It can be not only used for heat capacity measurements but can also be used for measuring other dynamic thermal properties such as thermal conductivity and diffusivity. The importance of this model stems from the fact that understanding thermal properties of biological cells is crucial in many biomedical applications, especially those related to biochemical reactions in biological systems. The model presented herein can also be used to characterize thermal properties of chemical samples and also show the potential of using BMCs in lab-on-a-chip compatible thermal characterization techniques. Further attempts will be dedicated to optimize the design and dimensions of cantilevers in order to improve the overall performance of a BMC and increase the sensitivity.
